# Case Report: Multiple brain abscess-like lesions with concurrent detection of *Nocardia amamiensis* and *Leishmania infantum* in cerebrospinal fluid

**DOI:** 10.3389/fimmu.2026.1881701

**Published:** 2026-09-30

**Authors:** Jiahao Liang, Gang Wang, Ruile Shen

**Affiliations:** Department of Neurology, The First Affiliated Hospital of Henan University of Science and Technology, Luoyang, Henan, China

**Keywords:** brain abscess-like lesions, *Leishmania infantum*, *Nocardia amamiensis*, targeted next-generation sequencing, visceral leishmaniasis

## Abstract

Central nervous system nocardiosis is rare and potentially life-threatening, and human infection due to *Nocardia amamiensis* has seldom been reported; central nervous system involvement in visceral leishmaniasis is likewise uncommon. We report a 69-year-old man who developed progressive impairment of consciousness and coma after recurrent fever. Brain magnetic resonance imaging revealed multiple abscess-like lesions in both cerebral hemispheres, the cerebellum, and the brainstem, accompanied by acute or subacute cerebral infarctions. The cerebrospinal fluid opening pressure was 300 mmH_2_O. The white blood cell count was 285 × 10^6^/L, with 81.9% polymorphonuclear cells; protein was 1.977 g/L and glucose was 2.34 mmol/L. MetaCAP analysis of cerebrospinal fluid concurrently detected *N. amamiensis* and *L. infantum*, and bone marrow smear demonstrated *Leishmania* amastigotes within macrophages. The patient tested negative for human immunodeficiency virus, and abdominal ultrasonography showed no hepatosplenomegaly. On the available evidence, visceral leishmaniasis was established and central nervous system nocardiosis was strongly suspected. Detection of *L. infantum* in cerebrospinal fluid supported possible central nervous system involvement, but did not establish a direct contribution to the brain abscess-like lesions. The patient received trimethoprim-sulfamethoxazole, imipenem-cilastatin, amikacin, and liposomal amphotericin B, but his condition continued to deteriorate. He was discharged against medical advice, and follow-up revealed that he had died. This case illustrates that sequencing-based detection is an important clue but cannot replace culture, pathogen-specific polymerase chain reaction, or evidence from lesion tissue.

## Introduction

Members of the genus *Nocardia* are aerobic, Gram-positive, partially acid-fast, branching filamentous bacteria. Nocardiosis occurs predominantly in individuals with impaired cell-mediated immunity and has a predilection for the lungs and central nervous system (CNS); hematogenous dissemination may produce solitary or multiple brain abscesses. Early diagnosis of CNS nocardiosis remains difficult because clinical and imaging findings are nonspecific, culture is time-consuming, and the yield of cerebrospinal fluid (CSF) culture is limited (1–3). Human infection due to *N. amamiensis* has rarely been reported, with previous cases involving mainly pulmonary, ocular, or disseminated disease (4, 5).

*Leishmania infantum* primarily parasitizes the mononuclear phagocyte system. Typical visceral leishmaniasis is characterized by prolonged fever, hepatosplenomegaly, and pancytopenia, although its clinical phenotype is not invariably typical (6). Locally acquired cases have continued to be reported in Henan Province in recent years (7), whereas human CNS involvement has rarely been described and uniform diagnostic criteria are lacking (8). Pathogen next-generation sequencing has expanded detection in culture-negative infections and for rare pathogens. Nevertheless, detection of multiple microorganisms in the same specimen does not establish that each is pathogenic; interpretation requires integration of specimen type, inflammatory response, histopathology, and orthogonal testing (9). We describe a patient with multiple brain abscess-like lesions in whom *N. amamiensis* and *L. infantum* were concurrently detected in CSF, focusing on the different levels of diagnostic evidence supported by the findings for each organism.

## Genomic testing methods

Bronchoalveolar lavage fluid (BALF) was analyzed using hybrid capture-based targeted next-generation sequencing (hc-tNGS). Nucleic acids were extracted with the MagPure Pathogen DNA/RNA Extraction Kit (Magen Biotechnology, Guangzhou, China). Reverse transcription and library construction were performed using the Reverse Transcription Kit and Library Construction Kit (KingCreate, Guangzhou, China), respectively. Following hybrid capture with the MetaCAP Pathogen Capture Metagenomic Assay Kit (KingCreate, Guangzhou, China), 100-bp single-end sequencing was performed on a NextSeq 550Dx platform (Illumina, San Diego, CA, United States). The panel covers 3,060 DNA and RNA pathogens, including bacteria, fungi, viruses, and parasites (10).

Cerebrospinal fluid was analyzed using MetaCAP hybrid capture-based targeted next-generation sequencing. Nucleic acids were extracted with the MasterPure DNA & RNA Extraction Kit (KingCreate, Guangzhou, China). RNA was reverse-transcribed into complementary DNA, after which libraries were constructed and subjected to probe capture. Subsequent 100-bp single-end sequencing was performed on a MiniSeq platform (Illumina, San Diego, CA, United States). The publicly reported panel covers 1,060 bacterial, 388 fungal, 1,402 viral, and 210 parasitic targets (11). Raw reads were quality filtered using fastp (v0.23.1), aligned to the human reference genome hg38 using BWA (v0.7.17-r1188), and depleted of human sequences. The remaining sequences were aligned against a pathogen reference database constructed from NCBI Genome.

The bone marrow aspirate also underwent high-throughput pathogen nucleic acid testing. Because this was a retrospective case and the available records did not preserve the specific reagents, sequencing instrument, or bioinformatic parameters, these details were not inferred. All sequencing findings were interpreted in conjunction with specimen type, clinical manifestations, imaging, conventional microbiologic testing, and bone marrow morphology. Detection of pathogen nucleic acid alone was not regarded as direct evidence of pathogenicity.

## Case description

A 69-year-old Han Chinese man who worked as a farmer had a history of hypertension, ischemic stroke, superficial gastritis, and diabetes mellitus. He tested negative for human immunodeficiency virus (HIV). He had no history of organ transplantation, malignancy, long-term glucocorticoid or immunosuppressive therapy, or other known immunosuppressive conditions.

In August 2024, the patient developed exertional dyspnea. In September 2025, when the exertional dyspnea worsened and jaw tremor developed, chest computed tomography (CT) was performed and showed emphysema and small areas of pulmonary infiltrate. Electromyography suggested diaphragmatic paralysis, and bulbar palsy was considered. In December 2025, he developed rhinorrhea, dizziness, and fever. The principal clinical events, investigations, and treatments are summarized in **Figure 1**.

**Figure 1:**
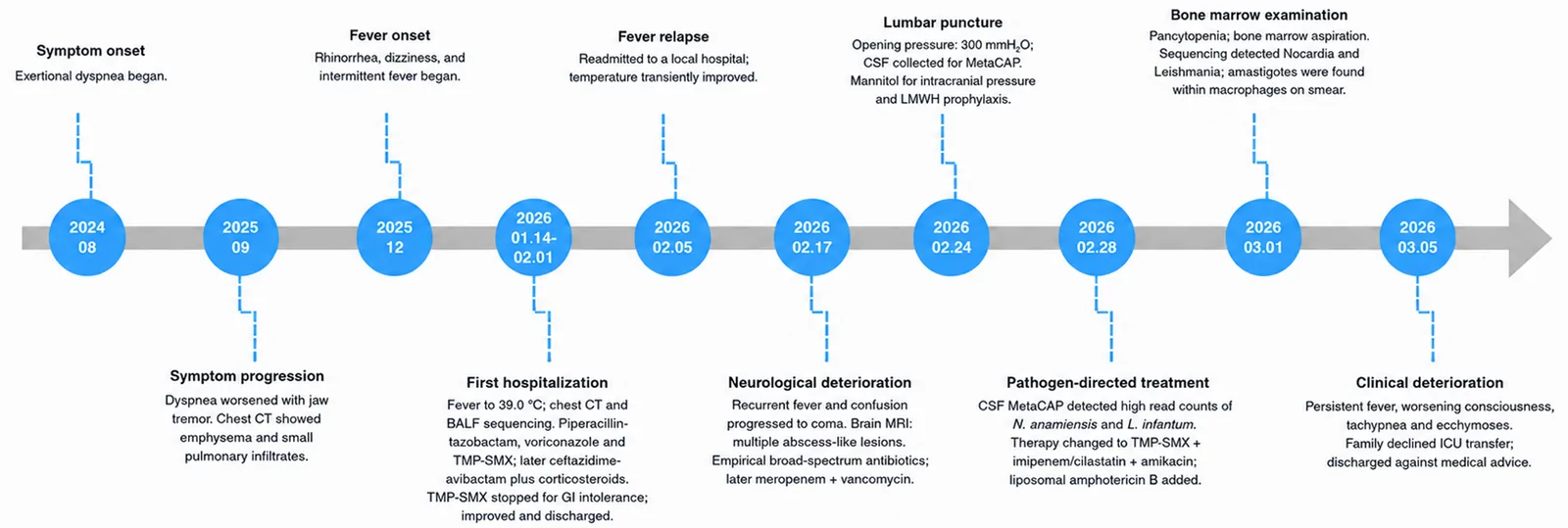
Clinical timeline. The timeline includes the onset of exertional dyspnea in August 2024, worsening exertional dyspnea with jaw tremor and chest CT in September 2025, the onset of rhinorrhea, dizziness, and fever in December 2025, and subsequent hospital admissions, specimen collection and reporting dates, changes in anti-infective therapy, and clinical outcome through March 2026.

On January 14, 2026, the patient was admitted with fever reaching 39.0 °C, chills, sore throat, fatigue, myalgia, and rhinorrhea. Chest CT on January 16 showed a pneumonic opacity with an air bronchogram in the posterior segment of the right upper lobe (**Figure 2A**). Follow-up chest CT on January 27 showed that the opacity had slightly decreased in size, with reduced surrounding infiltrates (**Figure 2B**). Subsequent high-throughput BALF testing detected *Klebsiella pneumoniae*, *Pneumocystis jirovecii*, *Aspergillus flavus*, Epstein-Barr virus (EBV), cytomegalovirus (CMV), and herpes simplex virus type 1 (HSV-1) (**Supplementary Table S1**). The patient received piperacillin-tazobactam, voriconazole, and trimethoprim-sulfamethoxazole (TMP-SMX) sequentially. Because the fever persisted, antibacterial therapy was changed to ceftazidime-avibactam, and a short course of prednisone acetate was administered from January 29 to February 1. TMP-SMX was discontinued after approximately 1 week because of gastrointestinal adverse effects. He was discharged on February 1 after his symptoms improved.

**Figure 2:**
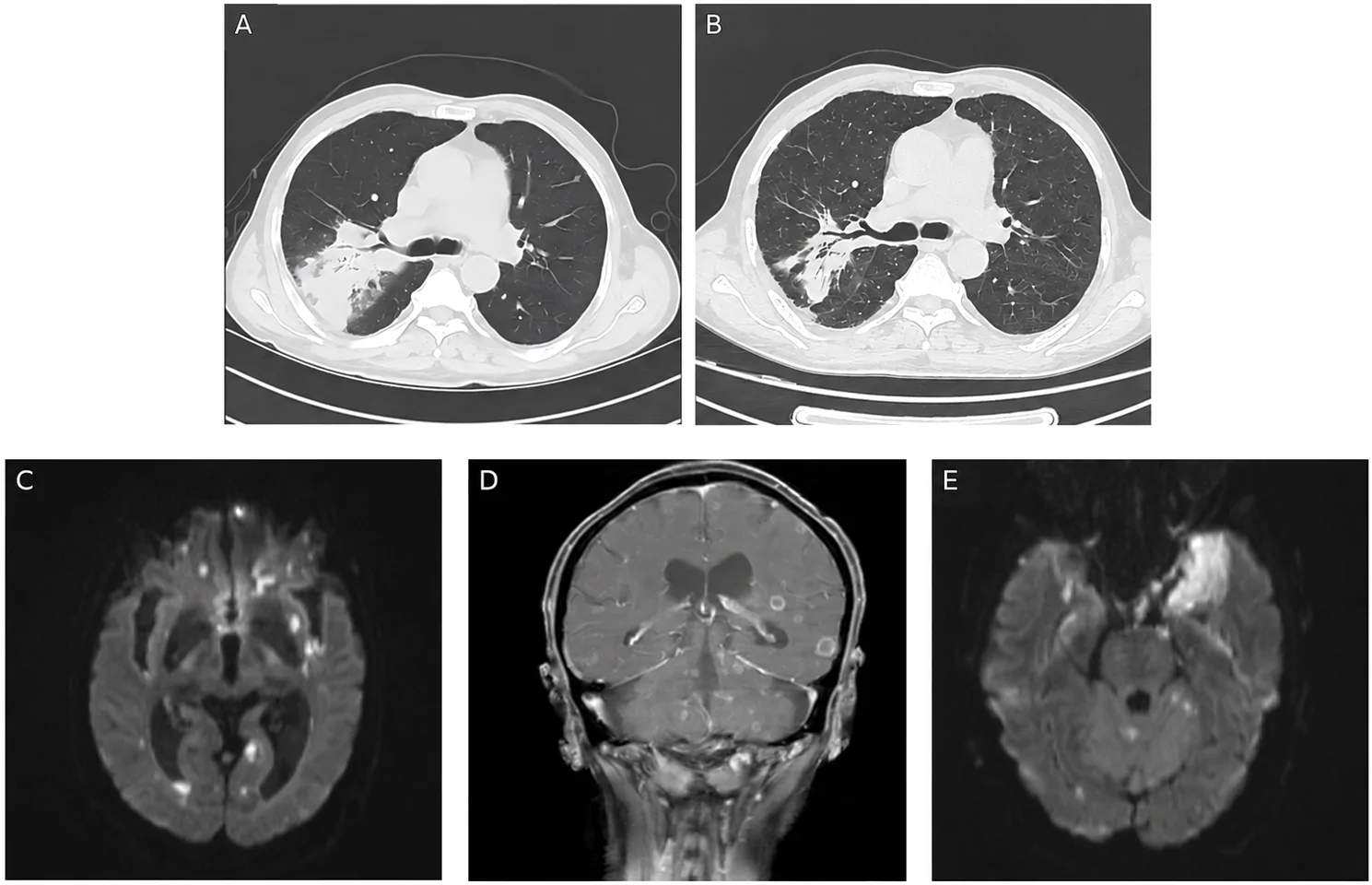
Chest computed tomography and brain magnetic resonance imaging. **(A)** Chest computed tomography on January 16, 2026, showed a pneumonic opacity with an air bronchogram in the posterior segment of the right upper lobe. **(B)** Follow-up chest computed tomography on January 27, 2026, showed that the right upper lobe opacity had slightly decreased in size, with reduced surrounding infiltrates. **(C, E)** Diffusion-weighted imaging from the brain MRI performed on February 17, 2026, showed multiple hyperintense foci in both cerebral hemispheres. **(D)** Contrast-enhanced magnetic resonance imaging from the same examination showed multiple ring- or nodular-enhancing lesions in both cerebral hemispheres, the cerebellum, and the brainstem.

Fever recurred on February 5, reaching 38.3 °C. His temperature temporarily decreased after treatment at a local hospital, but details of the anti-infective regimen were unavailable. On February 17, the fever recurred with confusion and subsequently progressed to coma, prompting transfer to our hospital. On admission, he was comatose, with coarse breath sounds and moist crackles in both lungs. Lymph node ultrasonography showed a single 25.9 mm × 8.7 mm hypoechoic lymph node in right cervical level V near the supraclavicular fossa. The node was fairly regular in shape, had relatively well-defined margins, and showed no appreciable blood flow on color Doppler imaging. No clearly abnormal lymph nodes were found in the left cervical region or either supraclavicular fossa. Abdominal ultrasonography showed no hepatomegaly or splenomegaly.

Brain magnetic resonance imaging (MRI) performed on February 17, 2026, revealed multiple abnormal enhancing and abscess-like lesions in both cerebral hemispheres, the cerebellum, and the brainstem. Diffusion-weighted imaging showed multiple hyperintense foci in both cerebral hemispheres, considered acute or subacute cerebral infarctions (**Figures 2C–E**). Cefoperazone-sulbactam was administered empirically for multiple brain abscesses and meningitis; because the response was limited, therapy was changed to meropenem plus vancomycin. Lumbar puncture on February 24 showed an opening pressure of 300 mmH_2_O. The CSF was colorless and slightly turbid, with a white blood cell count of 0.285 × 10^9^/L, comprising 0.233 × 10^9^/L polymorphonuclear cells (81.9%) and 0.052 × 10^9^/L mononuclear cells (18.1%). CSF protein was 1.977 g/L, glucose 2.34 mmol/L, chloride 109 mmol/L, and lactate dehydrogenase 239 U/L (**Supplementary Table S3**). Contemporaneous serum glucose, CSF culture, and cytology results were not retained in the available records; therefore, the CSF-to-serum glucose ratio could not be calculated.

On February 28, the CSF MetaCAP report identified 41,638 genus-level reads assigned to *Nocardia*, including 35,325 species-level reads for *N. amamiensis*, and 21,290 genus-level reads assigned to *Leishmania*, including 16,676 species-level reads for *L. infantum*. CMV (733 reads) and EBV (16 reads) were also detected (**Supplementary Table S2**). Treatment was changed to TMP-SMX, imipenem-cilastatin, and amikacin, with the addition of liposomal amphotericin B.

On March 1, a complete blood count showed pancytopenia: white blood cells, 2.94 × 10^9^/L; hemoglobin, 103 g/L; and platelets, 96 × 10^9^/L. High-throughput sequencing of the bone marrow aspirate detected 21 species-level reads for *N. amamiensis* and 480,149 for *L. infantum*, together with several viral sequences (**Supplementary Table S4**). Bone marrow smear demonstrated *Leishmania* amastigotes within macrophages (**Figure 3**), providing independent morphologic evidence of visceral leishmaniasis.

**Figure 3:**
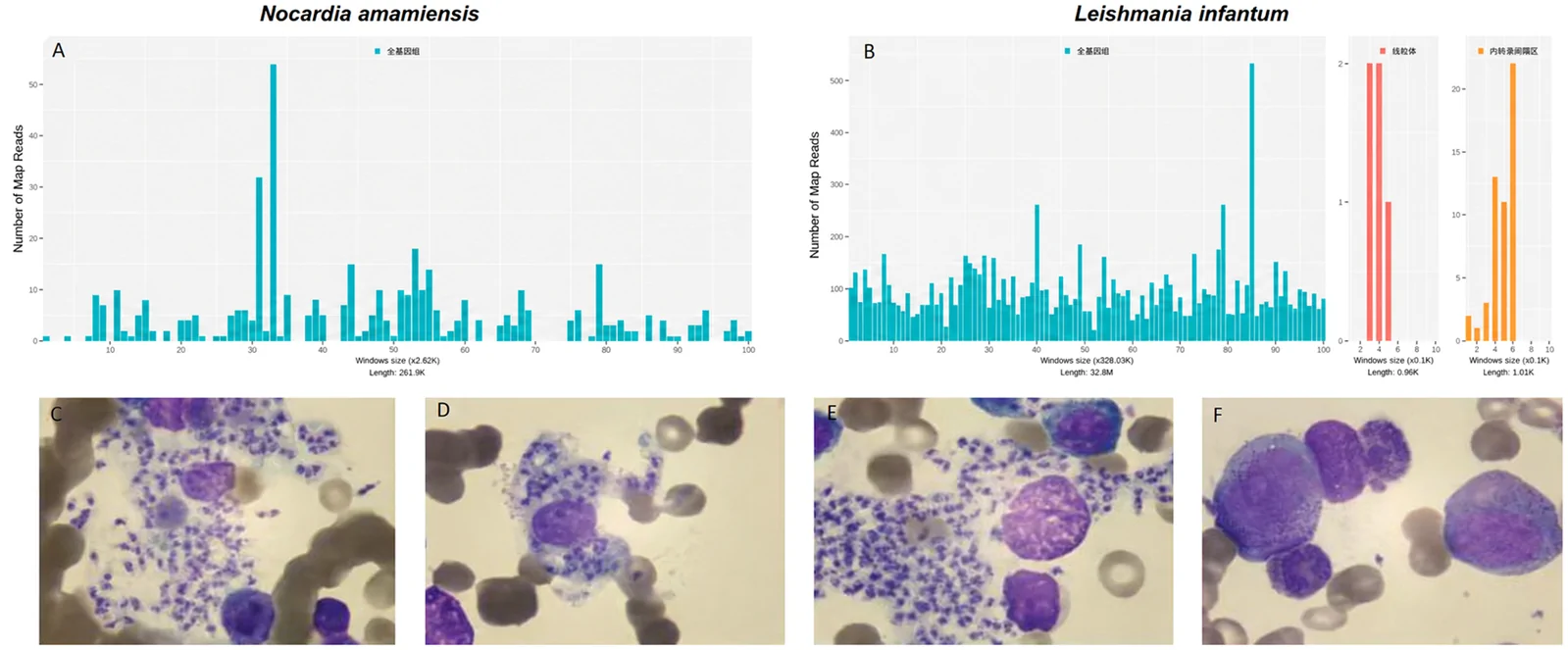
Pathogen detection and bone marrow morphologic evidence. **(A)** *N. amamiensis*: genomic coverage distribution. **(B)** *L. infantum*: genomic coverage distribution. **(C–F)** Bone marrow smears showing *Leishmania* amastigotes within macrophages.

On March 5, the patient continued to have intermittent fever, with worsening impairment of consciousness and tachypnea. While receiving oxygen by face mask, his heart rate was 134 beats/min, respiratory rate 35 breaths/min, and blood pressure 99/65 mmHg. Scattered ecchymoses were present over the right forehead and both lower extremities. Moist crackles were audible bilaterally, and nuchal rigidity of approximately two fingerbreadths was present, with positive signs of meningeal irritation. Coagulation testing showed a prothrombin time of 9.06 s, activated partial thromboplastin time of 44.80 s, fibrinogen of 4.56 g/L, and D-dimer of 10.40 mg/L fibrinogen-equivalent units. The family declined transfer to the intensive care unit and elected discharge against medical advice that day. At follow-up in June 2026, the family reported that the patient had died.

## Discussion

The most compelling evidence of CNS infection in this case concerned *N. amamiensis*. The multiple brain abscess-like lesions, signs of meningeal irritation, elevated intracranial pressure, neutrophil-predominant CSF inflammation, and 35,325 species-level reads were mutually supportive. However, read counts alone could not establish the respective contributions of the detected organisms to the brain lesions. Nocardial brain abscesses are often multiple, and CSF cultures are not invariably positive (2, 3). Nevertheless, no abscess tissue was obtained, and culture confirmation was unavailable. The case should therefore be described as highly suspected CNS nocardiosis rather than a confirmed *N. amamiensis* brain abscess. Because disseminated *N. amamiensis* infection with CNS involvement has previously been reported, this case should not be characterized as the first CNS infection worldwide (5).

The identification of amastigotes on bone marrow smear provided independent morphologic evidence of visceral leishmaniasis. The patient had prolonged fever and pancytopenia and resided in a previously endemic region. Although abdominal ultrasonography showed no hepatosplenomegaly, an atypical clinical phenotype does not outweigh the parasitologic evidence from bone marrow (6, 7). The patient was HIV-negative and had no history of organ transplantation or malignancy. Older age, diabetes, severe infection, and brief glucocorticoid exposure may have impaired host defenses; however, pancytopenia may also result from visceral leishmaniasis and severe infection and cannot itself be equated with severe immunosuppression. Human neuroleishmaniasis has rarely been reported, and uniform diagnostic criteria are lacking (8). Detection of *L. infantum* in cerebrospinal fluid supported possible central nervous system involvement, but did not establish a direct contribution to the brain abscess-like lesions.

Neither *N. amamiensis* nor *L. infantum*, which were later detected in CSF, was identified in BALF. Differences in pathogen burden between anatomical sites, sampling time, prior antimicrobial therapy, and assay workflow may account for this discordance, but their respective contributions could not be distinguished in this case (9, 10). Hybrid capture can increase the relative representation of target nucleic acids included in a panel, but results remain influenced by panel composition, sample preprocessing, and positivity thresholds (10, 11). Similarly, the multiple organisms detected in BALF and the low-level CMV and EBV signals in CSF could not be classified as true infection or contamination without culture, quantitative PCR, or other confirmatory testing.

Severe CNS nocardiosis generally requires a TMP-SMX-based regimen combined with one or more agents that achieve adequate CNS penetration, with subsequent adjustment according to antimicrobial susceptibility and clinical response; prolonged treatment is often required when the CNS is involved (12). Liposomal amphotericin B is an important treatment option for visceral leishmaniasis (13). After the CSF results became available, this patient received TMP-SMX, imipenem-cilastatin, amikacin, and liposomal amphotericin B. However, targeted treatment was initiated late and administered for only a short period, and the patient was discharged against medical advice while critically ill. Treatment efficacy therefore could not be assessed, and the death could not be attributed to any single pathogen or component of therapy.

The principal limitations of this case were the absence of brain abscess tissue or aspirate and the lack of Sanger sequencing or *in situ* testing. CSF culture, cytology, concurrent serum glucose, and some platform quality-control data were also unavailable. These limitations do not negate the value of pathogen sequencing as a diagnostic clue, but they restrict interpretation of the respective contributions of the two organisms to CNS disease.

## Conclusion

Bone marrow morphology established visceral leishmaniasis, while the clinical, imaging, and cerebrospinal fluid findings strongly supported central nervous system nocardiosis. Detection of *L. infantum* in cerebrospinal fluid supported possible central nervous system involvement, but its direct contribution to brain abscess formation was not established. When interpreting multi-pathogen sequencing findings, diagnoses supported by histopathology or culture should be distinguished from molecular signals that await validation.

## Data Availability

The original contributions presented in the study are included in the article/**Supplementary Material**. Further inquiries can be directed to the corresponding author.

## Supplementary material

Supplementary Table 1 Pathogen next-generation sequencing results for bronchoalveolar lavage fluid. BALF, bronchoalveolar lavage fluid; G-, Gram-negative; EBV, Epstein-Barr virus; CMV, cytomegalovirus; HSV-1, herpes simplex virus type 1; reads, sequence counts listed in the testing report. “High” and “moderate” were classifications in the original report; the available records did not disclose the underlying algorithm or thresholds. Pathogen loads were transcribed from the original report; their units and calculation method were not retained.

Supplementary Table 2 Cerebrospinal fluid MetaCAP results. CSF, cerebrospinal fluid; G+, Gram-positive; CMV, cytomegalovirus; EBV, Epstein-Barr virus; MetaCAP, the hybrid capture-based targeted next-generation sequencing platform used in this case. Relative abundance refers to the genus-level result. The “99% confidence” values were reported by the original platform and do not represent probabilities of clinical diagnosis; the available records did not disclose the underlying algorithm or thresholds.

Supplementary Table 3 Routine and biochemical cerebrospinal fluid findings. Results and reference ranges were transcribed from the original laboratory report. An em dash indicates that no reference range was reported or that the field was not applicable. CSF, cerebrospinal fluid; WBC, white blood cells; RBC, red blood cells; MN, mononuclear cells; PM, polymorphonuclear cells; ADA, adenosine deaminase; LDH, lactate dehydrogenase.

Supplementary Table 4 High-throughput pathogen nucleic acid testing of bone marrow aspirate. Values were transcribed from the original bone marrow aspirate report. An em dash indicates that a value was not provided or was not applicable. Relative abundance refers to the genus-level result. The “99% confidence” values were reported by the original platform and do not represent probabilities of clinical diagnosis; the available records did not disclose the underlying algorithm or thresholds. The original report did not detect special pathogens, fungi, antimicrobial resistance genes, or virulence genes; the anellovirus was categorized as a “putative human microbiome organism.” G+, Gram-positive; dsDNA, double-stranded DNA; ssDNA, single-stranded DNA; BK, BK polyomavirus; JC, JC polyomavirus; CMV, cytomegalovirus; HSV-1, herpes simplex virus type 1; EBV, Epstein-Barr virus. The original report listed 257 genus-level *Betapolyomavirus* reads, whereas the BK and JC species-level reads summed to 258; the values are retained as reported.
