# Supplementary material for: Case Report: Multiple brain abscess-like lesions with concurrent detection of Nocardia amamiensis and Leishmania infantum in cerebrospinal fluid
Source: Front Immunol. 2026 Sep 30;17:1881701. doi: 10.3389/fimmu.2026.1881701 (PMC13661340; doi:10.3389/fimmu.2026.1881701)
Supplement: Supplementary file 1 [file Table1.pdf]

Supplementary Table S1. Pathogen next-generation sequencing results for bronchoalveolar lavage fluid

Category	Genus	Genus-level reads	Species	Confidence	Species-level reads	Pathogen load
Bacteria (G-)	*Klebsiella*	121	*Klebsiella pneumoniae*	High	117	2.14×10^2
Fungi	*Pneumocystis*	486	*Pneumocystis jirovecii*	High	486	2.62×10^2
Fungi	*Aspergillus*	153	*Aspergillus flavus*	High	153	2.96×10^2
DNA virus	EBV	—	Human herpesvirus 4	High	202	7.81×10^2
DNA virus	CMV	—	Human herpesvirus 5	Moderate	93	1.53×10^2
DNA virus	HSV-1	—	Human herpesvirus 1	Moderate	35	<100

Note. BALF, bronchoalveolar lavage fluid; G-, Gram-negative; EBV, Epstein-Barr virus; CMV, cytomegalovirus; HSV-1, herpes simplex virus type 1; reads, sequence counts listed in the testing report.

“High” and “moderate” were classifications in the original report; the available records did not disclose the underlying algorithm or thresholds. Pathogen loads were transcribed from the original report; their units and calculation method were not retained.
