# Supplementary material for: Case Report: Multiple brain abscess-like lesions with concurrent detection of Nocardia amamiensis and Leishmania infantum in cerebrospinal fluid
Source: Front Immunol. 2026 Sep 30;17:1881701. doi: 10.3389/fimmu.2026.1881701 (PMC13661340; doi:10.3389/fimmu.2026.1881701)
Supplement: Supplementary file 2 [file Table2.pdf]

Supplementary Table S2. Cerebrospinal fluid MetaCAP results

Category	Genus	Genus-level reads	Relative abundance	Species	Species-level reads	Confidence
Bacteria (G+)	*Nocardia*	41,638	90.52%	*Nocardia amamiensis*	35,325	99%
DNA virus	*Cytomegalovirus*	733	75.17%	CMV	733	99%
DNA virus	*Lymphocryptovirus*	16	1.56%	EBV	16	99%
Parasite	*Leishmania*	21,290	100.00%	*Leishmania infantum*	16,676	99%

Note. CSF, cerebrospinal fluid; G+, Gram-positive; CMV, cytomegalovirus; EBV, Epstein-Barr virus; MetaCAP, the hybrid capture-based targeted next-generation sequencing platform used in this case. Relative abundance refers to the genus-level result.

The “99% confidence” values were reported by the original platform and do not represent probabilities of clinical diagnosis; the available records did not disclose the underlying algorithm or thresholds.
