# Supplementary material for: Case Report: Multiple brain abscess-like lesions with concurrent detection of Nocardia amamiensis and Leishmania infantum in cerebrospinal fluid
Source: Front Immunol. 2026 Sep 30;17:1881701. doi: 10.3389/fimmu.2026.1881701 (PMC13661340; doi:10.3389/fimmu.2026.1881701)
Supplement: Supplementary file 3 [file Table3.pdf]

Supplementary Table S3. Routine and biochemical cerebrospinal fluid findings

Category	Analyte	Abbreviation	Result	Unit	Reference range	Flag
Appearance	Color	—	Colorless	—	—	—
Appearance	Clarity	—	Slightly turbid	—	Clear	Abnormal
Appearance	Clot formation	—	Negative	—	—	—
Cell count	White blood cell count	WBC-BF	0.285	$\times10^9$/L	0-0.01	High
Cell count	Red blood cell count	RBC-BF	0	$\times10^{12}$/L	—	—
Cell count	Mononuclear cell count	MN#	0.052	$\times10^9$/L	—	—
Cell count	Polymorphonuclear cell count	PM#	0.233	$\times10^9$/L	—	—
Cell count	Mononuclear cell proportion	MN%	18.1	%	—	—
Cell count	Polymorphonuclear cell proportion	PM%	81.9	%	—	—
Biochemistry	Adenosine deaminase	ADA	7	U/L	0-8	Within range
Biochemistry	Lactate dehydrogenase	LDH	239	U/L	3-40	High
Biochemistry	Glucose	GLU	2.34	mmol/L	2.8-4.5	Low
Biochemistry	Potassium	K+	2.27	mmol/L	2.7-5.0	Low
Biochemistry	Sodium	Na+	142	mmol/L	136-145	Within range
Biochemistry	Chloride	Cl-	109	mmol/L	120-130	Low
Biochemistry	CSF protein	CSFPRO	1,977	mg/L	150-450	High

Note. Results and reference ranges were transcribed from the original laboratory report. An em dash indicates that no reference range was reported or that the field was not applicable.

CSF, cerebrospinal fluid; WBC, white blood cells; RBC, red blood cells; MN, mononuclear cells; PM, polymorphonuclear cells; ADA, adenosine deaminase; LDH, lactate dehydrogenase.
