# Supplementary material for: Case Report: Multiple brain abscess-like lesions with concurrent detection of Nocardia amamiensis and Leishmania infantum in cerebrospinal fluid
Source: Front Immunol. 2026 Sep 30;17:1881701. doi: 10.3389/fimmu.2026.1881701 (PMC13661340; doi:10.3389/fimmu.2026.1881701)
Supplement: Supplementary file 4 [file Table4.pdf]

Supplementary Table S4. High-throughput pathogen nucleic acid testing of bone marrow aspirate

Category	Genus	Genus-level reads	Relative abundance	Species/type/subtype	Species-level reads	Confidence
Bacteria (G+)	*Nocardia*	26	36.36%	*Nocardia amamiensis*	21	99%
DNA virus (dsDNA)	*Betapolyomavirus*	257	42.04%	Human polyomavirus 1 (BK polyomavirus)	233	99%
DNA virus (dsDNA)	*Betapolyomavirus*	257	42.04%	Human polyomavirus 2 (JC polyomavirus)	25	99%
DNA virus (dsDNA)	*Cytomegalovirus*	87	14.18%	Human cytomegalovirus (CMV)	87	99%
DNA virus (dsDNA)	*Simplexvirus*	83	13.53%	Herpes simplex virus 1 (HSV-1)	83	99%
DNA virus (dsDNA)	*Lymphocryptovirus*	16	2.60%	Epstein-Barr virus (EBV)	16	99%
Parasite	*Leishmania*	538,331	100.00%	*Leishmania infantum*	480,149	99%
Putative human microbiome organism (ssDNA)	*Alphatorquevirus*	166	—	Torque teno virus	166	—

Note. Values were transcribed from the original bone marrow aspirate report. An em dash indicates that a value was not provided or was not applicable. Relative abundance refers to the genus-level result. The “99% confidence” values were reported by the original platform and do not represent probabilities of clinical diagnosis; the available records did not disclose the underlying algorithm or thresholds.

The original report did not detect special pathogens, fungi, antimicrobial resistance genes, or virulence genes; the anellovirus was categorized as a “putative human microbiome organism.” G+, Gram-positive; dsDNA, double-stranded DNA; ssDNA, single-stranded DNA; BK, BK polyomavirus; JC, JC polyomavirus; CMV, cytomegalovirus; HSV-1, herpes simplex virus type 1; EBV, Epstein-Barr virus.

The original report listed 257 genus-level *Betapolyomavirus* reads, whereas the BK and JC species-level reads summed to 258; the values are retained as reported.
